# Air pollutants as modulators of mitochondrial quality control in cardiovascular disease

**DOI:** 10.14814/phy2.70118

**Published:** 2024-11-19

**Authors:** Kit Neikirk, Chanel Harris, Han Le, Ashton Oliver, Bryanna Shao, Kaihua Liu, Heather K. Beasley, Sydney Jamison, Jeanne A. Ishimwe, Annet Kirabo, Antentor Hinton

**Affiliations:** ^1^ Department of Molecular Physiology and Biophysics Vanderbilt University Nashville Tennessee USA; ^2^ Department of Anatomy of Cell Biology University of Iowa Iowa City Iowa USA; ^3^ Department of Medicine, Division of Clinical Pharmacology Vanderbilt University Medical Center Nashville Tennessee USA; ^4^ Vanderbilt Center for Immunobiology Nashville Tennessee USA; ^5^ Vanderbilt Institute for Infection, Immunology and Inflammation Nashville Tennessee USA; ^6^ Vanderbilt Institute for Global Health Nashville Tennessee USA

**Keywords:** cardiovascular disease, environment, mitochondria, pollutants, toxicology

## Abstract

It is important to understand the effects of environmental factors such as air pollution on mitochondrial structure and function, especially when these changes increase cardiovascular disease risk. Although lifestyle choices directly determine many mitochondrial diseases, increasingly, it is becoming clear that the structure and function of mitochondria may be affected by pollutants found in the atmosphere (e.g., gases, pesticides herbicide aerosols, or microparticles). To date, the role of such agents on mitochondria and the potential impact on cardiovascular fitness is neglected. Here we offer a review of airborne stressors and pollutants, that may contribute to impairments in mitochondrial function and structure to cause heart disease.

## 1 INTRODUCTION

Mitochondria are fundamental in eukaryotic cells, forming numerous phenotypes in response to their environment to carry out cellular functions, including energy production, calcium signaling, and regulation of apoptosis (Monzel et al., 2023). The emergence of heart failure has been associated with mitochondrial dysfunction (Bayeva et al., 2013; Chen et al., 2009). The heart's primary energy source is oxidative metabolism in the mitochondria, and it has long been believed that the key mechanism connecting mitochondrial dysfunction and contractile failure is the inability to produce and transport energy (Zhou & Tian, 2018). It is becoming more widely understood that mitochondria have a role in heart failure and cardiovascular diseases (CVD), beyond that of malfunctioning energy production (Hunter et al., 2016; Kiyuna et al., 2018; Liu et al., 2022). Mechanisms of mitochondrial dysfunction include increased mitochondrial Ca^2+^, increased mitochondrial reactive oxidative species (ROS) production, decreased mitochondrial membrane potential, and decreased ATP production (Hunter et al., 2016; Kiyuna et al., 2018; Liu et al., 2022). It is increasingly becoming clear that mitochondrial metabolism may be interrupted by certain environmental factors or chemicals, contributing to increasing mitochondrial age‐related dysfunction, a current hallmark of an aging heart (Barja, 2014).

Environmental factors include exposure to pollutants, chemicals, and lifestyle choices. These factors can alter mitochondrial function and structure through mechanisms including changes in biochemical pathways, mitochondrial morphology, and mitochondrial DNA (mtDNA). These are plentiful across multiple organisms; for example, in plants, aromatic hydrocarbons—which are known environmental pollutants—have been shown through transmission electron microscopy to cause fragmented mitochondria (Bayeva et al., 2013). This, in turn, affects contact sites (Bayeva et al., 2013), known as mitochondria endoplasmic reticulum contact sites (MERCs) which are formed to facilitate the exchange of ions, and intracellular signals, and to maintain calcium homeostasis (Chen et al., 2009). MERCs also aid the exchange of ROS, which helps cells balance antioxidants during oxidative stress (Ziegler et al., 2021). It has been shown that air‐borne pollutants may cause an increase in oxidative stress (Zhou & Tian, 2018). This can cause a deleterious cycle in which oxidative stress can exacerbate endoplasmic reticulum (ER) stress, in turn disrupting redox status and affecting the structure/function of MERCs (Cao & Kaufman, 2014). As previously reviewed, common mediators underlying pathophysiologic processes associated with CVDs, including hypertension, are both oxidative stress (Touyz et al., 2020) and ER stress (Balhara et al., 2024). Given this complex interdependence of mitochondrial quality control mechanisms, especially across aging (Hinton, Vue, et al., 2024), we examine how environmental factors in air pollution may increase the risk for CVDs.

## 2 CONCERNING TRENDS IN AIR POLLUTION AFFECTING CARDIOVASCULAR DISEASES

As previously reviewed, heart failure and other cardiovascular diseases (CVDs) have risen in prominence partially due to acute exposure to air pollution (Shah et al., 2013). Pollution causes around one in every nine deaths worldwide, with ambient air pollution, which includes potentially hazardous particulate matter arising from toxic chemical and industrial pollution, increasing in lethality as a consequence of urbanization (Fuller et al., 2022). Heart failure deaths and hospitalizations are both significantly linked to nitrogen dioxide, sulfur dioxide, and carbon monoxide concentrations (Shah et al., 2013). As global warming continues to occur, beyond only having an increasingly aged population, increased pollution may increase the global risk of heart failure (Shah et al., 2013). As previously reviewed, tolerance to hemorrhage can also be decreased in response to environmental stressors (Crandall et al., 2019). Even on a more fundamental level, heart rate variability may be altered by environmental factors (Uusitalo et al., 2007).

Other sources of air pollution include noise traffic, manufacturing, the production of electricity, wildfires, and even wood burner cooking. Smoking is one of the most prevalent indoor sources and poses a risk to smokers as well as the surrounding people. Another source of environmental pollution is noise. The primary cause of noise‐related health effects is road traffic noise. Traffic‐related noise pollution is becoming a bigger public health issue. A WHO expert panel concluded that traffic noise was linked to heart disease (Bhatnagar, 2006). The renin‐angiotensin‐aldosterone system is subsequently activated due to the perception of noise and the following cortical and sympathetic activation (Münzel et al., 2021). Stress hormones such as cortisol and catecholamines are produced. This pathway may eventually result in MI, heart failure, arterial hypertension, arrhythmia, and stroke if it is persistently present (American Heart Association, 2021). It may also cause the development of cardiovascular risk factors, blood clotting factor activation, and high blood pressure (Uusitalo et al., 2007).

Air pollution is a major health risk contributing to morbidity and excess mortality (Dahlquist et al., 2023). Globally, 45%–50% of excess fatalities are attributable to CVDs brought on by air pollution (de Bont et al., 2022; Lee et al., 2014). Long‐term exposure increases the risk of death. Elevations in air pollution have been linked to an increased risk of arrhythmia, heart failure, chronic coronary and peripheral artery disease, and acute coronary syndrome (Uusitalo et al., 2007), vulnerable individuals, such as the elderly or those with underlying medical issues, may be more prone to cardiovascular complications (e.g., heart attacks, strokes, arrhythmias, and heart failure) when exposed to short‐term air pollution (reviewed extensively in (de Bont et al., 2022; Lee et al., 2014; Lelieveld et al., 2019; Rajagopalan & Landrigan, 2021; Sagheer et al., 2024)). It was discovered that short‐term air pollution, such as ozone135 and particulate matter, is linked to out‐of‐hospital cardiac arrest (Dahlquist et al., 2023). Specifically, according to current research, air pollution may contribute to the onset and advancement of atherosclerosis, a condition in which plaque accumulates in the arterial walls and results in heart disease (Bhatnagar, 2006). Traffic noise and air pollution have been linked to changes in epigenetic DNA, which primes the tissues for modified inflammatory cascades and immune response modifications, according to a Swiss cohort research called SAPALDIA (Liu et al., 2007). Together, this demonstrates a prescient need to understand the metabolic mechanisms that underlie exacerbated risk of CVDs caused by air pollution.

## 3 AIR POLLUTION AS A FACTOR OF MITOCHONDRIAL DYSFUNCTION

**FIGURE 1:**
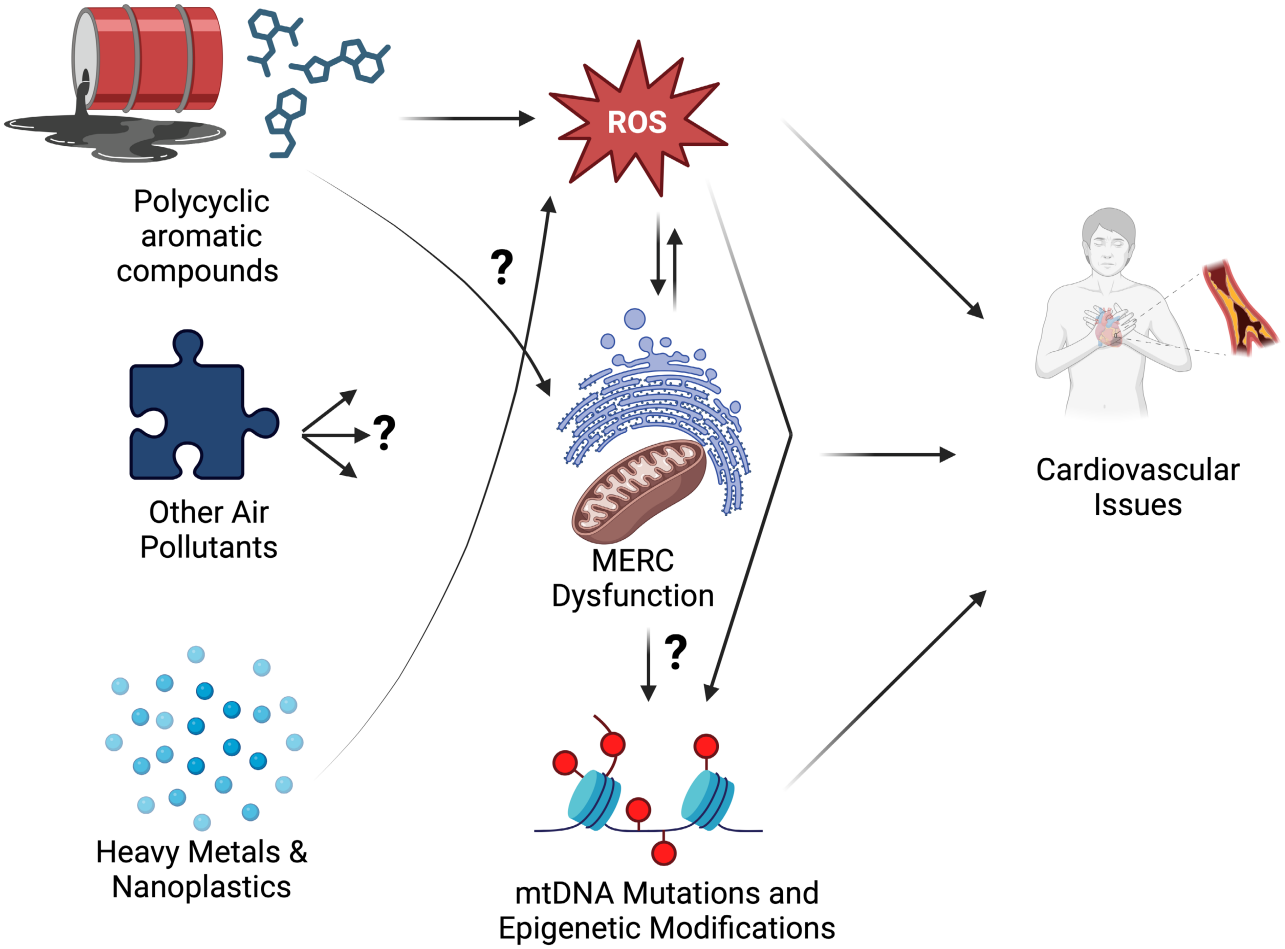
Environmental factors and other air pollution influencing mitochondria to affect cardiovascular disease, as well as considerations of other potential factors, such as MERC dynamics and mtDNA epigenetics, which require increased research due to their potential to interact further and contribute to cardiovascular complications.

In humans, while the mitochondrial role in heart failure is undisputed and extensively previously reviewed (Hinton, Claypool, et al., 2024; Zhou & Tian, 2018), the influence of pollution is an emergent area of research. This is all the more true as mitochondrial endpoints, such as epigenetic modifications, which are neglected in the heart, may lead to pollution‐dependent changes in the mitochondria (Wang et al., 2020). Thus, as heart failure and CVDs steadily cause large burdens on our healthcare systems, mechanisms of toxicity in the heart through mitochondria must be considered (Figure 1). Direct activation of the lung‐neural reflex arcs promotes neuronal activation and neuroinflammation, which connects pulmonary exposure to the consequences of air pollution on the brain and body (Münzel et al., 2021). Damage to the heart and brain is caused by oxidative stress mechanisms induced by air pollution (de Prado et al., 2018) (Figure 1). These mechanisms may also cause subsequent inflammation and gene activation, and they are mostly similar over a broad spectrum of various particles.

Mitochondrial structure has emerged to respond dynamically to stimuli, allowing for a wide range of mitochondrial phenotypes (Jenkins et al., 2024). For example, autophagosomes, which are linked to mitochondria structure through their contact sites and mitophagy (Neikirk, Vue, et al., 2023), alter in response to environmental exposure to heat (McCormick et al., 2022). While mitochondria are noted to change in 3D structure across the aging process in a tissue‐dependent manner (Vue et al., 2022), specifically how mitochondrial age‐linked structural change may be accelerated by environmental factors is still unknown. Some environmental factors may also have indirect effects. For example, organochlorine pesticides have been shown to affect mitochondrial calcium levels (Ko et al., 2020). Dysregulated calcium has in turn been shown to form abnormal mitochondrial structures such as nanotunnels, which are novel mitochondrial structures that arise during stress (Lavorato et al., 2017). Nanotunnels are slender double‐membrane projections connecting the matrix of nonadjacent mitochondria and facilitating intermitochondrial exchanges (Vincent et al., 2017, 2019). Recent data indicates that transport proteins and immobilized mitochondria produce mitochondrial nanotunnels (Vincent et al., 2017, 2019). Additionally, as a key component of cell‐to‐cell communication, nanotunnels may be compromised during mitochondria damage, thus implicated in cardiovascular disorders. Although nanotunnels functions are not well understood, nanotunnels have been noted to have increased appearance in patients who have a high degree of mtDNA mutations (Vincent et al., 2019). Given that the accumulation of mtDNA mutations is a hallmark of the aging process and linked to temperature, the potential role of nanotunnels, as well as their existence in response to certain environmental stressors demand further elucidation.

Beyond morphology, mitochondrial membrane potential is also affected secondary to oxidant stress incurred by environmental stressors (Barja, 2014). Recent research has elucidated that the folds of the inner mitochondrial membrane, cristae, have independent membrane potentials concomitant with differences in function (Naidoo & Naidoo, 2016). Therefore, there remains a gap in the literature regarding how cristae morphology and membrane potential may also be altered due to environmental stressors. Previously, a 3D reconstruction study comparing cardiac tissue mitochondria from young (3‐month‐old) and old (2‐year‐old) mice showed that aging had an impact on the morphology and dynamics of the mitochondria (Vue et al., 2023). Given that aged cardiac tissue has altered mitochondrial 3D structure (Vue et al., 2023), exposure to environmental stressors with age may lead to structurally linked bioenergetic changes in mitochondria leading to CVDs. Utilizing current techniques, including electron microscopy, with adequate rigor [as reviewed in (Marshall, Damo, & Hinton, 2023; Marshall, Neikirk, et al., 2023; Neikirk, Lopez, et al., 2023)], mitochondrial structure and ultrastructure must be evaluated in response to air pollutants.

Air pollution is increasingly understood to be linked to mitochondrial methylation patterns (Breton et al., 2019; Wang et al., 2020), yet less clear is how unique mitochondria structures may be influenced by air pollution. In general, previous research has shown that environmental pollutants such as aluminum oxide particles (AlNPs) can alter mitochondrial dynamics and induce oxidative stress (Mirshafa et al., 2018) (Figure 1). Air pollutants have been shown to cause oxidative stress and inflammation in human olfactory mucosal cells (Chew et al., 2020). Beyond this, air pollution can decrease mitochondrial oxygen efficiency as well as alter mtDNA (Breton et al., 2019), yet the underlying mechanisms for these alterations require further elucidation. Given that MERCs are known to be modulated by ER stress proteins and alter mitochondria efficiency (Giacomello & Pellegrini, 2016), these contact sites may also be important to study as a potential modulator of mitochondrial perseverance. Notably, beyond calcium signaling, several MERC proteins are implicated in ROS generation (Resende et al., 2022). MERCs are highly sensitive to environmental factors and when exposed to toxins, mitochondrial membranes undergo compositional shifts, which then affects organelle contracts and alterations in lipid metabolism. Notably, MERCs play important roles in heart disease, where their calcium homeostatic roles maintain mitochondria fusion/fission dynamics to prevent cardiac dysfunction and heart failure (Wu et al., 2017). One potential avenue to explore is how MERCs impacted by air pollution may be restored through methods such as artificial tethering (Nichtová et al., 2023).

## 4 ENVIRONMENTAL FACTORS RELATED TO AIR POLLUTION

Beyond these traditional air pollutants and consideration of ambient air pollution, there is a need for future studies to consider other pollutants that may be airborne, in turn affecting mitochondrial metabolism and quality control, contributing to CVDs. The next sections discuss a selection of these factors which will remain nascent areas of research.

### 4.1 Temperature and heat stress

As global warming occurs, increasingly organelles may undergo adaptations to different temperatures. As previously reviewed, climate change‐driven increases in air pollution and heat exposure have synergistic effects, exacerbating each one's pernicious effects (Anenberg et al., 2020). Specifically, a study found that, regardless of rural or urban locations, prolonged heatwaves increased air pollutants, with some, such as ozone, increasing more than 50% (Kalisa et al., 2018). Temperature has been shown to affect mitochondria at the protein level and mtDNA, including historically where colder environments have resulted in lower mitochondrial diversity as seen through mtDNA (Balloux et al., 2009). Mitochondria, which are responsible for producing aerobic energy in cells, are greatly impacted by temperature. Temperature plays a role in redox balance, ATP generation, and respiration, among other mitochondrial processes (Lau et al., 2020). In older individuals, temperature rises have been linked to an increased level of mitochondrial DNA damage (Peng et al., 2017) Modifications in temperature can affect mitochondrial function, which can influence the capacity of mitochondria to sustain energy balance. For instance, even when respiratory activity is limited, short‐term exposure to temperatures exceeding 43°C can cause respiratory complexes to become unstable (Moreno‐Loshuertos et al., 2023). However, other studies have suggested that the high temperature of mitochondria at baseline, owing to their enzymatic activity, is resistant to external metabolic stresses (Terzioglu et al., 2023). While in ecotherms the effects of global temperature changes have been considered (Im, 2023), it remains unclear if small but chronic increases in bodily temperature of humans may affect metabolic activity, beyond only changes owing to exacerbated air pollution.

### 4.2 Polyfluoroalkyl substances and toxicants

Several key airborne toxicants must be explored further in the context of CVDs. As previously reviewed, the nuclear epigenome and mitochondria engage in cross‐talking in response to environmental toxicology (Weinhouse, 2017). In particular, this can result in toxicant‐induced effects on mtDNA mutagenesis over the lifespan including through increased de novo and altered mtDNA mutation frequencies (Weinhouse, 2017), with other studies demonstrating that the effects of environmental toxicants may be ameliorated through reduction of mtDNA content (Luz & Meyer, 2016).

Per‐ and polyfluoroalkyl substances (PFAS) are common in consumer items including nonstick cookware, which can lead to increased exposure across the lifespan (Jenkins et al., 2024). As reviewed, while PFAS are most commonly considered in the context of their long‐term chemical stability within bodies of water, PFAS and early precursor PFAS (e.g., volatile species) may also be found and transmitted in atmospheric particulate matter (Evich et al., 2022). Notably, a recent study found that PFAS were ubiquitously detected in the particulate matter from several Asian cities, with forms such as perfluorooctanoic acid having airborne concentrations as high as 77.9 pg/m^3^ (Lin et al., 2020). PFAS are well understood to cause hepatotoxicity, but increasingly it is understood this may be through mitochondrial‐dependent mechanisms involving the induction of oxidative stress and apoptosis (Jiao et al., 2021). While these effects are most frequently observed in adverse pregnancy outcomes (Jiao et al., 2021), previous reviews have highlighted the role of PFAS in inducing cardiac toxicity, hypertension, and dyslipidemia, all risk factors of heart failure (Wen et al., 2023). Notably, Liu et al. have recently shown that in Zebrafish PFAS exposure increases energy expenditure antecedent to increased glycolysis and disruption of underlying metabolic homeostasis (Liu et al., 2023). Beyond only suggesting a plausible link through which PFAS exposure may increase the risk of heart failure, this study also shows an interface between lipid metabolism, through β‐oxidation, and mitochondria may govern the development of metabolic dysfunctions due to PFAS (Liu et al., 2023). This underscores the importance of increased research that understands PFAS‐dependent changes in lipids and mitochondria, such as through investigating mitochondria‐lipid contact sites, which mediate lipid metabolism (Benador et al., 2018). As current restrictions have caused a shift to the manufacture and emission of short‐chain PFAS (Lin et al., 2020), the risks of these forms of PFAS in particulate matter are worth further investigation.

### 4.3 Heavy metals

Heavy metals are known to play a role in differences in energy metabolism, underscoring their potential to induce toxicity through mitochondrial alterations (Yang et al., 2020). Heavy metals can be inhaled, penetrate through skin, or enter the digestive tract through consumption (Witkowska et al., 2021). While some metals are needed in the body to maintain homeostasis, excessive amounts have been shown to have damaging effects (Jaishankar et al., 2014). Smoking‐specific brands of cigarettes have also been proven to contain Cr, Ni, and Cd which when inhaled, accumulate in the body (Ashraf, 2012), as well as contribute to air pollution through secondhand smoking. In particular, hexavalent chromium (Cr(VI)), a common occupational toxicant, is a pulmonary carcinogen that can act as an ambient airborne particulate matter (Huang et al., 2014). A study within New Jersey found higher soluble Cr(VI) concentrations in the summer, suggesting a potential link between temperature and ambient air pollution (Huang et al., 2014). There has been evidence showing that the longer individuals work at industrial jobs the greater risk they have for heavy metal toxification (Abd Elnabi et al., 2023). Cr(VI) impacts the generation of ROS while also interfering with the AMPK/PGC1‐α pathway biogenesis by affecting the biogenesis of the mitochondria (Yang et al., 2020). Chronic exposure to hexavalent chromium in rats—akin to that which occurs across aging in many industrial jobs can result in heart dysfunction through inhibition of Sesn2 and subsequent mitochondrial dysfunction (Yang et al., 2021). However, many wide meta‐analyses of Cr(VI) in humans focus on the outcomes of liver diseases or other non‐cardiovascular outcomes as a result of hexavalent chromium (Chakraborty et al., 2022; Ray, 2016), neglecting its potential influence on CVD. Additionally, as previously reviewed, the relationship between the heart and liver is complicated (El Hadi et al., 2020), however, risk factors of heart failure arising from hepatotoxicity remain unclear. Other heavy metals, such as vanadium and cadmium, can both accumulate within the mitochondria and cause cardiac mitochondria to have a buildup in reactive oxygen species (Soares et al., 2008), thus underscoring the need to explicate the relation between cumulative life span heavy metal exposure in airborne particulate matter and mitochondrial‐dependent CVDs.

### 4.4 Nanoplastics and new toxicants

Outside of common toxicants, numerous other toxicants must be explored in the context of potential effects on mitochondria. One emerging airborne toxicant of interest are micro‐ and nano‐plastics, which have reported atmospheric concentrations as high as 1583 items m^−3^, a burden that is only expected to increase with the continued increase in global plastic production (reviewed in (Bhat et al., 2023; Le et al., 2023; Luo et al., 2024)). As previously reviewed, micro‐ and nano‐plastics are known to be common risk factors for cardiovascular disorders, including heart failure, through cardiotoxicity and promotion of thrombosis (Bostan et al., 2016; Liang et al., 2024). Nanoplastics break down into tiny molecules that can enter the cardiovascular system by penetrating veins. Other reviews have specified some of the other multifaceted effects on mitochondria caused by micro‐ and nano‐plastics, including ER stress and inflammation (Lee et al., 2022; Wu et al., 2024). Specifically, polyethylene nanoplastics, in Zebrafish embryos, can cause cardiotoxicity through the generation of oxidative stress (Sun et al., 2021) implicating a mitochondrial role (Bostan et al., 2016; Liang et al., 2024). Of particular interest, amino‐functionalized nanoplastics in human umbilical vein endothelial cells resulted, alongside oxidative stress, decreased mitochondria membrane potential (Fu et al., 2022). Nanoplastics have also been associated with reproductive toxicity in a male murine model through mtDNA mislocalization, upregulation of fission proteins, and downregulation of fusion proteins, together causing fission in mitochondria and leading to apoptosis (Zhao, Xie, et al., 2024). However, it is unclear if these changes are translatable to human models.

Besides nanoplastics, new pollutants that should be considered include flame retardant, stove‐top‐generated gases, and other emerging environmental contaminants. Increasingly, the influence of these toxicants on mitochondria will be able to be better elucidated due to new screening tools that allow for the evaluation of mitochondrial toxicity (Zheng et al., 2021). However, studies that investigate the role of age‐dependent accumulation of these toxicants as a potential risk of heart failure are important. Beyond this, while many of these toxicants have been studied for their role in oxidative stress, literature remains limited which investigates how they may alter mitochondria in other mechanisms, such as structure and contact sites with lipid droplets and other organelles (Reddam et al., 2022).

### 4.5 Organic pollutants, pesticides, and other organic chemicals

Organic pollutants, pesticides, and hydrocarbons are often neglected when discussing mitochondrial function. Previous studies have shown the outsized impact of persistent organic pollutants, including through human serum levels (Patterson Jr Donald et al., 2009). Individuals can be exposed to these pollutants, in part, through airborne spreading, such as spreading of pesticides (Seiber & Cahill, 2022). Across various organic pollutants (e.g., dibenzo‐p‐dioxins and dibenzofurans, polychlorinated biphenyls, polybrominated diphenyl ethers, and organochlorine pesticides) a multitude of studies have reported concentrations within the air across many geographical regions, which may in turn contribute to the pathological outcomes of organic pollutants (Al Dine et al., 2015; de la Torre et al., 2016; Polkowska et al., 2000; Pribylova et al., 2012; Seiber & Cahill, 2022). Previous reviews have discussed how pesticides may commonly affect mitochondria metabolism through alterations in oxidative capacity and impairment of complex activity (Zolkipli‐Cunningham & Falk, 2017). Yet, less clear is how other mitochondrial quality control mechanisms may be altered. As previously discussed and reviewed (Reddam et al., 2022), a key biomarker of mitochondria alteration is mtDNA. However, whether various organic pollutants can either increase or decrease mtDNA copy number, with the exact functional implications of these changes in the context of heart failure remaining unclear. Thus, ROS may be a better marker of alterations.

The buildup of ROS is generally seen as a hallmark of aging, and while data is conflicting, has been considered a significant cause of overall mitochondrial reduced function across age (Barja, 2014). Notably, increased ROS is a hallmark of many mitochondrial dysfunctional states prompted by environmental factors including heavy metals and metalloids—both of which reduce antioxidant activity and increase inflammation to cause oxidative stress (Blajszczak & Bonini, 2017). Oxidative stress is a well‐known factor in disease states (Monzel et al., 2023), including being linked to ischemic damage (Kuzmiak‐Glancy et al., 2022). Previous research has been conducted on the effect of ROS triggered by ozone exposure being linked to the amyloidogenic pathway to promote the likelihood of Alzheimer's Disease (Kiyuna et al., 2018). Interestingly, it was found that polycyclic aromatic compounds (PAC) increase levels of ROS in neural cells which later may lead to inducing apoptosis signaling in that cell (Sarma et al., 2017). Additionally, it has been found that with repeated exposure to PAC, oxidative damage, and lipid peroxidation are activated (Jeng et al., 2011). One study found that specifically phenanthrene, which is a PAC, can induce ER stress, and oxidative stress (Wang et al., 2022). However, it is not just aromatic compounds that are the source of dysfunction. Both found in pesticides, paraquat, and rotenone have been shown to induce oxidative stress and inhibit mitochondrial complex I (Chen et al., 2021; Khalil et al., 2015; Tanner et al., 2011). Notably, a key mechanism in which mitochondria confer protection against pollutant‐mediated prolonged periods of high ROS is through maintaining mtDNA integrity (Liu et al., 2022).

An interesting future avenue is changes in mitochondria methylation pattern changes. As previously reviewed, mitochondrial methylation is an important mechanism that carries effects on epigenetic modification of mitochondria (Byun & Baccarelli, 2014). Plaat et al., have linked global DNA methylation with occupational exposure to pesticides (van der Plaat et al., 2018), yet few studies have done the same for mtDNA. Similarly, the compensatory mechanisms for organic compounds impacting these systems remain unclear; for example, past studies have shown that in response to the ROS generated by organic compounds, autophagy, and apoptosis may be positive changes that prevent long‐term damage (Rainey et al., 2017). It remains unclear if similar mechanisms exist for methylation to undo epigenetic changes caused by environmental stress.

A clear future avenue is aromatic hydrocarbons, such as 2,3,7,8‐Tetrachlorodibenzo‐p‐dioxin, which may cause irregular mitochondrial signaling. For aromatic hydrocarbons, their activation is primarily mediated by cytochrome P450 enzymes, with activation then causing interaction with xenobiotic receptors (Zhao et al., 2021). Specifically, in an aromatic hydrocarbon receptor‐dependent manner, these compounds generate oxidative stress and lead to xenobiotic metabolism as well as activation of other mitochondrial pathways which can cause apoptosis (Zhao et al., 2021). This same xenobiotic pathway is of relevance, as it is through a similar mechanism in which heavy metals such as Cadmium, cause PINK1/Parkin‐dependent mitophagy (Zhang et al., 2021). However, future studies must elucidate the specific receptors, beyond the aryl hydrocarbon receptor (Chavan & Krishnamurthy, 2012), that may modulate these interactions. Beyond this, it is unclear if mitophagy reductions or mechanisms to reduce oxidative stress may undo these changes. Of relevance, given the lipid‐like formation of polycyclic aromatic hydrocarbons (Chavan & Krishnamurthy, 2012), an interesting future study will be to better understand how this similarity may interact with the mitochondrial membrane potential. Notably, while ROS and apoptosis are intrinsically linked to CVDs (Poznyak et al., 2020; Touyz et al., 2020), recent research has also shown that myocardial infarction may be attenuated by mitochondrial membrane potential improvements (Sun & Yang, 2017), suggesting a potential mechanism by which PACs act upon the cardiovascular system.

### 4.6 Endocrine disruptors and bisphenol A

A new point of current research is the study of how changes in cholesterol, testosterone, and, importantly, estrogen levels affect mitochondrial function (Jia et al., 2014). Some of the roles of mitochondria are linked to testosterone: as testosterone decreases the generation of ROS and the creation of energy and the operation of the mitochondrial electron transport chain depend on testosterone (Tostes et al., 2016); by promoting the transcription of mitochondrial proteins, testosterone elevates the composition of mitochondria (Toro‐Urrego et al., 2016). Additionally, optic atrophy protein 1 (OPA1), a nuclear‐encoded mitochondrial protein that controls MERCs and mitochondrial stability, is induced to express by testosterone (Pronsato et al., 2020). While the role of testosterone is of undoubted importance in the context of regulating environmental factors, of particular interest is estrogen regulation mitochondria because generally, the high levels of estrogen associated with the period of fertility in women are understood to serve protective roles against age‐related diseases including those that are cardiovascular (Parisi et al., 2023).

Estrogen, a steroid hormone that functions in female reproduction has been linked to regulating cholesterol levels, bone mass, and the cardiovascular system (Xiang et al., 2021). Cholesterol functions to maintain the fluidity of the plasma membrane and excess is directed by the cell to the mitochondria and ER, where ROS is produced (Li & Pfeffer, 2016; Szabo et al., 2023). Studies have recognized that excess ROS involving the mitochondria can lead to the development of CVD (Poznyak et al., 2020). Fertile women with exposure to endogenous estrogens have a decreased risk for CHD and atherosclerotic disease. (Yang et al., 2020) Specifically, 17β‐estradiol, which increases during menstruation, has been studied to understand estrogen's role in the regulation of mitochondria in cardiac cells (Luo & Kim, 2016). Men who have decreased amounts of estrogen have been shown to have decreased vasodilation and increased oxidative stress which can lead to CVDs (Javed et al., 2023). While there has been evidence showing this link between fertile women and decreased risk for CVDs these risks increase postmenopausal (Parisi et al., 2023).

Thus, several endocrine disruptors' mitochondrial‐mediated cardiac functions are worth examining further for their roles in air pollution. As extensively reviewed before, there are a multitude of air pollutants associated with endocrine disruption, including some of the heavy metals and pesticides discussed in prior sections (Darbre, 2018). Some of the major ones that are prevalent in the air and have cardiovascular are Phthalates and bisphenol A (BPA) (Darbre, 2018). Phthalate exposure has previously been reviewed to be closely linked to CVDs through mitochondrial quality control mechanisms, including oxidative stress and structural changes (Kabekkodu et al., 2024). Additionally, recent results have demonstrated that in neurotoxicity, phthalates may interrupt MERCs by impairing the Mfn2‐PERK Axis (Zhao, Chang, et al., 2024). This suggests a secondary mechanism may exist through which endocrine disruptors alter MERC formations, which remains poorly studied.

BPA is another example of an endocrine disruptor that is becoming more prominent in the environment (Hafezi & Abdel‐Rahman, 2019). It can be found in plastics, water supply pipes, and importantly, can reside in water, dust, and air (Bisphenol, n.d.). Notably, while BPA is found more plentifully indoors than outdoors in developed countries, it has the capability for long‐range atmospheric transport (Vasiljevic & Harner, 2021). BPA can interact with hormone receptors and alter their usual signaling pathway. Studies in female mice have shown that exposure to BPA can also cause irregular heartbeats and interfere with intracellular calcium regulation further impacting heart function (Fonseca et al., 2022). While metabolism‐disrupting chemicals are typically considered in the context of neonatal effects, understanding how this different altered regulation of steroids alters cardiac fitness is therefore deeply important in the future.

## 5 FUTURE PERSPECTIVE

As we have previously reviewed, the field of mitochondrial research is undergoing constant metamorphosis, with ethnicity‐related differences in insulin stimulation (Neikirk, Kabugi, et al., 2024), mitochondrial transplantation (Neikirk, Stephens, et al., 2024), and broader insight into quality control mechanisms of mitochondria (Hinton, Claypool, et al., 2024; Jenkins et al., 2024), all representing areas of research also pertinent to understanding air pollution. Another key avenue to explore further in the future is mtDNA. mtDNA mutations and diseases are increasingly becoming an issue, with age‐associated heteroplasmy highlighted as a key factor in heart failure (Elorza & Soffia, 2021). Notably, a study by Wang et al., has shown that for individuals in a highly populated area, there is a greater likelihood of mtDNA mutations (Wang et al., 2020). However, how mechanisms by which mtDNA modulates mitochondria protection against environmental factors remain unclear. For example, it remains controversial if mtDNA methylation, a negative epigenetic effector for risk factors (including cancer), occurs in response to factors such as air pollutants (Hunter et al., 2016). Beyond this, it is still unclear what other factors may modulate mtDNA's sensitivity to changes in chronic ROS or other mitochondrial alterations caused by environmental factors. This makes mtDNA a similarly important future avenue for study (Figure 1). For example, studies should look at how mtDNA copied numbers are controlled and if ultrastructural changes aid in this regulation (Monzel et al., 2023). Another avenue would be to elucidate how mtDNA density further differs among certain cell types and modulates tissue‐dependent responses to air pollution (Monzel et al., 2023).

## 6 CONCLUSION

In tandem, it is clear that certain environmental stressors related to air pollutants cause increases in mtDNA heteroplasmy, ROS buildup, and dysregulated mitochondria structure and contact sites. More research must be conducted on environmental stressors to develop further new therapeutic approaches against their potential role in increasing the risk of CVDs. This makes it pertinent to gain a better understanding of often neglected dynamics that modulate these functions.

## AUTHOR CONTRIBUTIONS

Kit Neilkirk: Writing and figures. Chanel Harris: Writing and edits. Han Le: Writing and edits. Ashton Oliver: Edits. Bryanna Shao: Edits. Kaihua Liu: Writing and edits. Heather K. Beasley: Writing and edits. Sydney Jamison: Writing and edits. Jeanne A. Ishimwe: Writing and edits. Annet Kirabo: Conceptualization, Writing and edits. Antentor Hinton Jr: Conceptualization, writing, edits, and figures.

## CONFLICT OF INTEREST STATEMENT

The authors have no conflicts of interest to declare.

## ETHICS STATEMENT

None.

## References

[phy270118-bib-0001] Abd Elnabi, M. K. , Elkaliny, N. E. , Elyazied, M. M. , Azab, S. H. , Elkhalifa, S. A. , Elmasry, S. , Mouhamed, M. S. , Shalamesh, E. M. , Alhorieny, N. A. , Abd Elaty, A. E. , Elgendy, I. M. , Etman, A. E. , Saad, K. E. , Tsigkou, K. , Ali, S. S. , Kornaros, M. , & Mahmoud, Y. A.‐G. (2023). Toxicity of heavy metals and recent advances in their removal: A review. Toxics, 11, 580. 10.3390/toxics11070580 37505546 PMC10384455

[phy270118-bib-0002] Al Dine, E. J. , Mokbel, H. , Elmoll, A. , Massemin, S. , Vuilleumier, S. , Toufaily, J. , Hanieh, T. , & Millet, M. (2015). Concomitant evaluation of atmospheric levels of polychlorinated biphenyls, organochlorine pesticides, and polycyclic aromatic hydrocarbons in Strasbourg (France) using pine needle passive samplers. Environmental Science and Pollution Research, 22, 17850–17859. 10.1007/s11356-015-5030-5 26162446

[phy270118-bib-0003] American Heart Association . (2021). Elevated stress hormones linked to higher risk of high blood pressure and heart events [Online]. http://newsroom.heart.org/news/elevated‐stress‐hormones‐linked‐to‐higher‐risk‐of‐high‐blood‐pressure‐and‐heart‐events [30 May 2024]

[phy270118-bib-0004] Anenberg, S. C. , Haines, S. , Wang, E. , Nassikas, N. , & Kinney, P. L. (2020). Synergistic health effects of air pollution, temperature, and pollen exposure: A systematic review of epidemiological evidence. Environmental Health, 19, 130. 10.1186/s12940-020-00681-z 33287833 PMC7720572

[phy270118-bib-0005] Ashraf, M. W. (2012). Levels of heavy metals in popular cigarette brands and exposure to these metals via smoking. The Scientific World Journal, 2012, 729430. 10.1100/2012/729430 22489199 PMC3320036

[phy270118-bib-0006] Balhara, M. , Neikirk, K. , Marshall, A. , Hinton, A. , & Kirabo, A. (2024). Endoplasmic reticulum stress in hypertension and salt sensitivity of blood pressure. Current Hypertension Reports, 26, 273–290. 10.1007/s11906-024-01300-9 38602583 PMC11166838

[phy270118-bib-0007] Balloux, F. , Handley, L.‐J. L. , Jombart, T. , Liu, H. , & Manica, A. (2009). Climate shaped the worldwide distribution of human mitochondrial DNA sequence variation. Proceedings of the Royal Society B: Biological Sciences, 276, 3447–3455. 10.1098/rspb.2009.0752 PMC2817182 19586946

[phy270118-bib-0008] Barja, G. (2014). The mitochondrial free radical theory of aging. Progress in Molecular Biology and Translational Science, 127, 1–27. 25149212 10.1016/B978-0-12-394625-6.00001-5

[phy270118-bib-0009] Bayeva, M. , Gheorghiade, M. , & Ardehali, H. (2013). Mitochondria as a therapeutic target in heart failure. Journal of the American College of Cardiology, 61, 599–610. 10.1016/j.jacc.2012.08.1021 23219298 PMC3594689

[phy270118-bib-0010] Benador, I. Y. , Veliova, M. , Mahdaviani, K. , Petcherski, A. , Wikstrom, J. D. , Assali, E. A. , Acín‐Pérez, R. , Shum, M. , Oliveira, M. F. , Cinti, S. , Sztalryd, C. , Barshop, W. D. , Wohlschlegel, J. A. , Corkey, B. E. , Liesa, M. , & Shirihai, O. S. (2018). Mitochondria bound to lipid droplets have unique bioenergetics, composition, and dynamics that support lipid droplet expansion. Cell Metabolism, 27, 869–885.e6. 10.1016/j.cmet.2018.03.003 29617645 PMC5969538

[phy270118-bib-0011] Bhat, M. A. , Gedik, K. , & Gaga, E. O. (2023). Atmospheric micro (nano) plastics: Future growing concerns for human health. Air Quality, Atmosphere and Health, 16, 233–262. 10.1007/s11869-022-01272-2 PMC9574822 36276170

[phy270118-bib-0012] Bhatnagar, A. (2006). Environmental cardiology: studying mechanistic links between pollution and heart disease. Circulation Research, 99(7), 692–705. 10.1161/01.res.0000243586.99701.cf 17008598

[phy270118-bib-0013] Bisphenol A (BPA) [Online]. [date unknown]. https://www.niehs.nih.gov/health/topics/agents/sya‐bpa/index.cfm [10 May 2023]

[phy270118-bib-0014] Blajszczak, C. , & Bonini, M. G. (2017). Mitochondria targeting by environmental stressors: Implications for redox cellular signaling. Toxicology, 391, 84–89. 10.1016/j.tox.2017.07.013 28750850 PMC5939563

[phy270118-bib-0015] Bostan, H. B. , Rezaee, R. , Valokala, M. G. , Tsarouhas, K. , Golokhvast, K. , Tsatsakis, A. M. , & Karimi, G. (2016). Cardiotoxicity of nano‐particles. Life Sciences, 165, 91–99. 10.1016/j.lfs.2016.09.017 27686832

[phy270118-bib-0016] Breton, C. V. , Song, A. Y. , Xiao, J. , Kim, S.‐J. , Mehta, H. H. , Wan, J. , Yen, K. , Sioutas, C. , Lurmann, F. , Xue, S. , Morgan, T. E. , Zhang, J. , & Cohen, P. (2019). Effects of air pollution on mitochondrial function, mitochondrial DNA methylation, and mitochondrial peptide expression. Mitochondrion, 46, 22–29. 10.1016/j.mito.2019.04.001 30980914 PMC6506186

[phy270118-bib-0017] Byun, H.‐M. , & Baccarelli, A. A. (2014). Environmental exposure and mitochondrial epigenetics: Study design and analytical challenges. Human Genetics, 133, 247–257. 10.1007/s00439-013-1417-x 24402053 PMC4351743

[phy270118-bib-0018] Cao, S. S. , & Kaufman, R. J. (2014). Endoplasmic reticulum stress and oxidative stress in cell fate decision and human disease. Antioxidants & Redox Signaling, 21, 396–413. 10.1089/ars.2014.5851 24702237 PMC4076992

[phy270118-bib-0019] Chakraborty, R. , Renu, K. , Eladl, M. A. , El‐Sherbiny, M. , Elsherbini, D. M. A. , Mirza, A. K. , Vellingiri, B. , Iyer, M. , Dey, A. , & Valsala, G. A. (2022). Mechanism of chromium‐induced toxicity in lungs, liver, and kidney and their ameliorative agents. Biomedicine & Pharmacotherapy, 151, 113119. 10.1016/j.biopha.2022.113119 35613529

[phy270118-bib-0020] Chavan, H. , & Krishnamurthy, P. (2012). Polycyclic aromatic hydrocarbons (PAHs) mediate transcriptional activation of the ATP binding cassette transporter ABCB6 gene via the aryl hydrocarbon receptor (AhR) *. Journal of Biological Chemistry, 287, 32054–32068. 10.1074/jbc.M112.371476 22761424 PMC3442536

[phy270118-bib-0021] Chen, J. , Su, Y. , Lin, F. , Iqbal, M. , Mehmood, K. , Zhang, H. , & Shi, D. (2021). Effect of paraquat on cytotoxicity involved in oxidative stress and inflammatory reaction: A review of mechanisms and ecological implications. Ecotoxicology and Environmental Safety, 224, 112711. 10.1016/j.ecoenv.2021.112711 34455184

[phy270118-bib-0022] Chen, L. , Gong, Q. , Stice, J. P. , & Knowlton, A. A. (2009). Mitochondrial OPA1, apoptosis, and heart failure. Cardiovascular Research, 84, 91–99. 10.1093/cvr/cvp181 19493956 PMC2741347

[phy270118-bib-0023] Chew, S. , Lampinen, R. , Saveleva, L. , Korhonen, P. , Mikhailov, N. , Grubman, A. , Polo, J. M. , Wilson, T. , Komppula, M. , Rönkkö, T. , Gu, C. , Mackay‐Sim, A. , Malm, T. , White, A. R. , Jalava, P. , & Kanninen, K. M. (2020). Urban air particulate matter induces mitochondrial dysfunction in human olfactory mucosal cells. Particle and Fibre Toxicology, 17, 18. 10.1186/s12989-020-00352-4 32487172 PMC7268298

[phy270118-bib-0024] Crandall, C. G. , Rickards, C. A. , & Johnson, B. D. (2019). Impact of environmental stressors on tolerance to hemorrhage in humans. American Journal of Physiology. Regulatory, Integrative and Comparative Physiology, 316, R88–R100. 10.1152/ajpregu.00235.2018 30517019 PMC6397352

[phy270118-bib-0025] Dahlquist, M. , Frykman, V. , Hollenberg, J. , Jonsson, M. , Stafoggia, M. , Wellenius, G. A. , & Ljungman, P. L. S. (2023). Short‐term ambient air pollution exposure and risk of out‐of‐hospital cardiac arrest in Sweden: A Nationwide Case‐Crossover Study. Journal of the American Heart Association, 12, e030456. 10.1161/JAHA.123.030456 37818697 PMC10727387

[phy270118-bib-0026] Darbre, P. D. (2018). Overview of air pollution and endocrine disorders. International Journal of General Medicine, 11, 191–207. 10.2147/IJGM.S102230 29872334 PMC5973437

[phy270118-bib-0027] de Bont, J. , Jaganathan, S. , Dahlquist, M. , Persson, Å. , Stafoggia, M. , & Ljungman, P. (2022). Ambient air pollution and cardiovascular diseases: An umbrella review of systematic reviews and meta‐analyses. Journal of Internal Medicine, 291, 779–800. 10.1111/joim.13467 35138681 PMC9310863

[phy270118-bib-0028] de la Torre, A. , Sanz, P. , Navarro, I. , & Martínez, M. Á. (2016). Time trends of persistent organic pollutants in spanish air. Environmental Pollution, 217, 26–32. 10.1016/j.envpol.2016.01.040 26843029

[phy270118-bib-0029] de Prado, B. P. , Mercader, E. M. H. , Pujol, J. , Sunyer, J. , & Mortamais, M. (2018). The effects of air pollution on the brain: A review of studies interfacing environmental epidemiology and neuroimaging. Current Environmental Health Reports, 5, 351–364. 10.1007/s40572-018-0209-9 30008171 PMC6132565

[phy270118-bib-0030] El Hadi, H. , Di Vincenzo, A. , Vettor, R. , & Rossato, M. (2020). Relationship between heart disease and liver disease: A two‐way street. Cells, 9, 567. 10.3390/cells9030567 32121065 PMC7140474

[phy270118-bib-0031] Elorza, A. A. , & Soffia, J. P. (2021). mtDNA heteroplasmy at the core of aging‐associated heart failure. An integrative view of OXPHOS and mitochondrial life cycle in cardiac mitochondrial physiology. Frontiers in Cell and Developmental Biology, 9, 625020. 10.3389/fcell.2021.625020 33692999 PMC7937615

[phy270118-bib-0032] Evich, M. G. , Davis, M. J. B. , McCord, J. P. , Acrey, B. , Awkerman, J. A. , Knappe, D. R. U. , Lindstrom, A. B. , Speth, T. F. , Tebes‐Stevens, C. , Strynar, M. J. , Wang, Z. , Weber, E. J. , Henderson, W. M. , & Washington, J. W. (2022). Per‐ and polyfluoroalkyl substances in the environment. Science, 375, eabg9065. 10.1126/science.abg9065 35113710 PMC8902460

[phy270118-bib-0033] Fonseca, M. , Lorigo, M. , & Cairrao, E. (2022). Endocrine‐disrupting effects of bisphenol A on the cardiovascular system: A review. JoX, 12, 181–213. 10.3390/jox12030015 PMC9326625 35893265

[phy270118-bib-0034] Fu, Y. , Fan, M. , Xu, L. , Wang, H. , Hu, Q. , & Jin, Y. (2022). Amino‐functionalized polystyrene Nano‐plastics induce mitochondria damage in human umbilical vein endothelial cells. Toxics, 10, 215. 10.3390/toxics10050215 35622629 PMC9145670

[phy270118-bib-0035] Fuller, R. , Landrigan, P. J. , Balakrishnan, K. , Bathan, G. , Bose‐O'Reilly, S. , Brauer, M. , Caravanos, J. , Chiles, T. , Cohen, A. , Corra, L. , Cropper, M. , Ferraro, G. , Hanna, J. , Hanrahan, D. , Hu, H. , Hunter, D. , Janata, G. , Kupka, R. , Lanphear, B. , … Yan, C. (2022). Pollution and health: A progress update. The Lancet Planetary Health, 6, e535–e547. 10.1016/S2542-5196(22)00090-0 35594895

[phy270118-bib-0036] Giacomello, M. , & Pellegrini, L. (2016). The coming of age of the mitochondria–ER contact: A matter of thickness. Cell Death and Differentiation, 23, 1417–1427. 10.1038/cdd.2016.52 27341186 PMC5072433

[phy270118-bib-0037] Hafezi, S. A. , & Abdel‐Rahman, W. M. (2019). The endocrine disruptor bisphenol A (BPA) exerts a wide range of effects in carcinogenesis and response to therapy. Current Molecular Pharmacology, 12, 230–238. 10.2174/1874467212666190306164507 30848227 PMC6864600

[phy270118-bib-0039] Hinton, A. , Claypool, S. M. , Neikirk, K. , Senoo, N. , Wanjalla, C. N. , Kirabo, A. , & Williams, C. R. (2024). Mitochondrial structure and function in human heart failure. Circulation Research, 135, 372–396. 10.1161/CIRCRESAHA.124.323800 38963864 PMC11225798

[phy270118-bib-0040] Hinton, A. O. , Vue, Z. , Scudese, E. , Neikirk, K. , Kirabo, A. , & Montano, M. (2024). Mitochondrial heterogeneity and crosstalk in aging: Time for a paradigm shift? Aging Cell, 23(10), e14296. 39188058 10.1111/acel.14296 PMC11464123

[phy270118-bib-0041] Huang, L. , Yu, C. H. , Hopke, P. K. , Lioy, P. J. , Buckley, B. T. , Shin, J. Y. , & Fan, Z. T. (2014). Measurement of soluble and total hexavalent chromium in the ambient airborne particles in New Jersey. Aerosol and Air Quality Research, 14, 1939–1949. 10.4209/aaqr.2013.10.0312 26120324 PMC4480920

[phy270118-bib-0042] Hunter, W. G. , Kelly, J. P. , McGarrah, R. W. , Khouri, M. G. , Craig, D. , Haynes, C. , Ilkayeva, O. , Stevens, R. D. , Bain, J. R. , Muehlbauer, M. J. , Newgard, C. B. , Felker, G. M. , Hernandez, A. F. , Velazquez, E. J. , Kraus, W. E. , & Shah, S. H. (2016). Metabolomic profiling identifies novel circulating biomarkers of mitochondrial dysfunction differentially elevated in heart failure with preserved versus reduced ejection fraction: Evidence for shared metabolic impairments in clinical heart failure. Journal of the American Heart Association, 5, e003190. 10.1161/JAHA.115.003190 27473038 PMC5015273

[phy270118-bib-0043] Im, S. (2023). Ectotherm mitochondrial economy and responses to global warming. Acta Physiologica (Oxford, England), 237, e13950. 10.1111/apha.13950 36790303

[phy270118-bib-0044] Jaishankar, M. , Tseten, T. , Anbalagan, N. , Mathew, B. B. , & Beeregowda, K. N. (2014). Toxicity, mechanism and health effects of some heavy metals. Interdisciplinary Toxicology, 7, 60–72. 10.2478/intox-2014-0009 26109881 PMC4427717

[phy270118-bib-0045] Javed, A. , Ravi, P. C. , Bilal Delvi, S. , Faraz Hussain, I. , Acosta, G. , Iqbal, W. , Krishnamaneni, V. , Alasaadi, S. , Pradhan, S. , Vashisht, R. , & Modi, S. (2023). The relationship between myocardial infarction and estrogen use: A literature review. Cureus, 15(9), e46134. 37900417 10.7759/cureus.46134 PMC10612533

[phy270118-bib-0046] Jeng, H. A. , Pan, C.‐H. , Diawara, N. , Chang‐Chien, G.‐P. , Lin, W.‐Y. , Huang, C.‐T. , Ho, C.‐K. , & Wu, M.‐T. (2011). Polycyclic aromatic hydrocarbon‐induced oxidative stress and lipid peroxidation in relation to immunological alteration. Occupational and Environmental Medicine, 68, 653–658. 10.1136/oem.2010.055020 21126960

[phy270118-bib-0047] Jenkins, B. C. , Neikirk, K. , Katti, P. , Claypool, S. M. , Kirabo, A. , McReynolds, M. R. , & Hinton, A. (2024). Mitochondria in disease: Changes in shapes and dynamics. Trends in Biochemical Sciences, 49, 346–360. 10.1016/j.tibs.2024.01.011 38402097 PMC10997448

[phy270118-bib-0048] Jia, G. , Aroor, A. R. , & Sowers, J. R. (2014). Estrogen and mitochondria function in cardiorenal metabolic syndrome. Progress in Molecular Biology and Translational Science, 127, 229–249. 10.1016/B978-0-12-394625-6.00009-X 25149220 PMC4318630

[phy270118-bib-0049] Jiao, X. , Liu, N. , Xu, Y. , & Qiao, H. (2021). Perfluorononanoic acid impedes mouse oocyte maturation by inducing mitochondrial dysfunction and oxidative stress. Reproductive Toxicology, 104, 58–67. 10.1016/j.reprotox.2021.07.002 34246765 PMC8477654

[phy270118-bib-0050] Kabekkodu, S. P. , Gladwell, L. R. , & Choudhury, M. (2024). The mitochondrial link: Phthalate exposure and cardiovascular disease. Biochimica et Biophysica Acta (BBA) – Molecular Cell Research, 1871, 119708. 10.1016/j.bbamcr.2024.119708 38508420

[phy270118-bib-0051] Kalisa, E. , Fadlallah, S. , Amani, M. , Nahayo, L. , & Habiyaremye, G. (2018). Temperature and air pollution relationship during heatwaves in Birmingham, UK. Sustainable Cities and Society, 43, 111–120. 10.1016/j.scs.2018.08.033

[phy270118-bib-0052] Khalil, W. K. B. , Assaf, N. , ElShebiney, S. A. , & Salem, N. A. (2015). Neuroprotective effects of bee venom acupuncture therapy against rotenone‐induced oxidative stress and apoptosis. Neurochemistry International, 80, 79–86. 10.1016/j.neuint.2014.11.008 25481089

[phy270118-bib-0053] Kiyuna, L. A. , Albuquerque, R. P. , Chen, C.‐H. , Mochly‐Rosen, D. , & Ferreira, J. C. B. (2018). Targeting mitochondrial dysfunction and oxidative stress in heart failure: Challenges and opportunities. Free Radical Biology and Medicine, 129, 155–168. 10.1016/j.freeradbiomed.2018.09.019 30227272 PMC6309415

[phy270118-bib-0054] Ko, E. , Choi, M. , & Shin, S. (2020). Bottom‐line mechanism of organochlorine pesticides on mitochondria dysfunction linked with type 2 diabetes. Journal of Hazardous Materials, 393, 122400. 10.1016/j.jhazmat.2020.122400 32135367

[phy270118-bib-0055] Kuzmiak‐Glancy, S. , Glancy, B. , & Kay, M. W. (2022). Ischemic damage to every segment of the oxidative phosphorylation cascade elevates ETC driving force and ROS production in cardiac mitochondria. American Journal of Physiology. Heart and Circulatory Physiology, 323, H499–H512. 10.1152/ajpheart.00129.2022 35867709 PMC9448280

[phy270118-bib-0056] Lau, G. Y. , Cox, G. K. , Stieglitz, J. D. , Benetti, D. D. , & Grosell, M. (2020). Temperature sensitivity differs between heart and red muscle mitochondria in mahi‐mahi (Coryphaena hippurus). Scientific Reports, 10, 14865. 10.1038/s41598-020-71741-0 32913250 PMC7484784

[phy270118-bib-0057] Lavorato, M. , Iyer, V. R. , Dewight, W. , Cupo, R. R. , Debattisti, V. , Gomez, L. , De la Fuente, S. , Zhao, Y.‐T. , Valdivia, H. H. , Hajnóczky, G. , & Franzini‐Armstrong, C. (2017). Increased mitochondrial nanotunneling activity, induced by calcium imbalance, affects intermitochondrial matrix exchanges. Proceedings of the National Academy of Sciences, 114, E849–E858. 10.1073/pnas.1617788113 PMC5293110 28096415

[phy270118-bib-0058] Le, V.‐G. , Nguyen, M.‐K. , Nguyen, H.‐L. , Lin, C. , Hadi, M. , Hung, N. T. Q. , Hoang, H.‐G. , Nguyen, K. N. , Tran, H.‐T. , Hou, D. , Zhang, T. , & Bolan, N. S. (2023). A comprehensive review of micro‐ and nano‐plastics in the atmosphere: Occurrence, fate, toxicity, and strategies for risk reduction. Science of the Total Environment, 904, 166649. 10.1016/j.scitotenv.2023.166649 37660815

[phy270118-bib-0059] Lee, B.‐J. , Kim, B. , & Lee, K. (2014). Air pollution exposure and cardiovascular disease. Toxicology Research, 30, 71–75. 10.5487/TR.2014.30.2.071 PMC4112067 25071915

[phy270118-bib-0060] Lee, S. E. , Yi, Y. , Moon, S. , Yoon, H. , & Park, Y. S. (2022). Impact of micro‐ and nanoplastics on mitochondria. Metabolites, 12, 897. 10.3390/metabo12100897 36295799 PMC9612075

[phy270118-bib-0061] Lelieveld, J. , Klingmüller, K. , Pozzer, A. , Pöschl, U. , Fnais, M. , Daiber, A. , & Münzel, T. (2019). Cardiovascular disease burden from ambient air pollution in Europe reassessed using novel hazard ratio functions. European Heart Journal, 40, 1590–1596. 10.1093/eurheartj/ehz135 30860255 PMC6528157

[phy270118-bib-0062] Li, J. , & Pfeffer, S. R. (2016). Lysosomal membrane glycoproteins bind cholesterol and contribute to lysosomal cholesterol export. eLife, 5, e21635. 10.7554/eLife.21635 27664420 PMC5068966

[phy270118-bib-0063] Liang, J. , Ji, F. , Abdullah, A. L. B. , Qin, W. , Zhu, T. , Tay, Y. J. , Li, Y. , & Han, M. (2024). Micro/nano‐plastics impacts in cardiovascular systems across species. Science of the Total Environment, 942, 173770. 10.1016/j.scitotenv.2024.173770 38851343

[phy270118-bib-0064] Lin, H. , Taniyasu, S. , Yamazaki, E. , Wei, S. , Wang, X. , Gai, N. , Kim, J. H. , Eun, H. , Lam, P. K. S. , & Yamashita, N. (2020). Per‐ and Polyfluoroalkyl substances in the air particles of Asia: Levels, seasonality, and size‐dependent distribution. Environmental Science & Technology, 54, 14182–14191. 10.1021/acs.est.0c03387 33156616

[phy270118-bib-0065] Liu, L.‐J. , Curjuric, I. , Keidel, D. , Heldstab, J. , Künzli, N. , Bayer‐Oglesby, L. , Ackermann‐Liebrich, U. , & Schindler, C. (2007). Characterization of source‐specific air pollution exposure for a large population‐based swiss cohort (SAPALDIA). Environmental Health Perspectives, 115, 1638–1645. 10.1289/ehp.10177 18007997 PMC2072852

[phy270118-bib-0066] Liu, M. , Lv, J. , Pan, Z. , Wang, D. , Zhao, L. , & Guo, X. (2022). Mitochondrial dysfunction in heart failure and its therapeutic implications. Frontiers in Cardiovascular Medicine, 9, 945142. 10.3389/fcvm.2022.945142 36093152 PMC9448986

[phy270118-bib-0067] Liu, Y. , Liu, S. , Huang, J. , Liu, Y. , Wang, Q. , Chen, J. , Sun, L. , & Tu, W. (2023). Mitochondrial dysfunction in metabolic disorders induced by per‐ and polyfluoroalkyl substance mixtures in zebrafish larvae. Environment International, 176, 107977. 10.1016/j.envint.2023.107977 37244004

[phy270118-bib-0068] Luo, D. , Chu, X. , Wu, Y. , Wang, Z. , Liao, Z. , Ji, X. , Ju, J. , Yang, B. , Chen, Z. , Dahlgren, R. , Zhang, M. , & Shang, X. (2024). Micro‐ and nano‐plastics in the atmosphere: A review of occurrence, properties and human health risks. Journal of Hazardous Materials, 465, 133412. 10.1016/j.jhazmat.2023.133412 38218034

[phy270118-bib-0069] Luo, T. , & Kim, J. K. (2016). The role of estrogen and estrogen receptors on cardiomyocytes: An overview. Canadian Journal of Cardiology, 32, 1017–1025. 10.1016/j.cjca.2015.10.021 26860777 PMC4853290

[phy270118-bib-0070] Luz, A. L. , & Meyer, J. N. (2016). Effects of reduced mitochondrial DNA content on secondary mitochondrial toxicant exposure in Caenorhabditis elegans. Mitochondrion, 30, 255–264. 10.1016/j.mito.2016.08.014 27566481 PMC5023498

[phy270118-bib-0071] Marshall, A. G. , Damo, S. M. , & Hinton, A. (2023). Revisiting focused ion beam scanning electron microcopy. Trends in Biochemical Sciences, 48, 585–586. 10.1016/j.tibs.2023.02.005 36990957

[phy270118-bib-0072] Marshall, A. G. , Neikirk, K. , Stephens, D. C. , Vang, L. , Vue, Z. , Beasley, H. K. , Crabtree, A. , Scudese, E. , Lopez, E. G. , Shao, B. , Krystofiak, E. , Rutledge, S. , Davis, J. , Murray, S. A. , Damo, S. M. , Katti, P. , & Hinton, A. (2023). Serial block face‐scanning electron microscopy as a burgeoning technology. Advanced Biology, 7(8), 2300139. 10.1002/adbi.202300139 PMC10950369 37246236

[phy270118-bib-0073] McCormick, J. J. , Côté, M. D. , King, K. E. , McManus, M. K. , Goulet, N. , Dokladny, K. , Moseley, P. L. , & Kenny, G. P. (2022). Autophagic response to exercise in peripheral blood mononuclear cells from young men is intensity‐dependent and is altered by exposure to environmental heat. American Journal of Physiology. Regulatory, Integrative and Comparative Physiology, 323, R467–R482. 10.1152/ajpregu.00110.2022 35993558

[phy270118-bib-0074] Mirshafa, A. , Nazari, M. , Jahani, D. , & Shaki, F. (2018). Size‐dependent neurotoxicity of aluminum oxide particles: A comparison between nano‐ and micrometer size on the basis of mitochondrial oxidative damage. Biological Trace Element Research, 183, 261–269. 10.1007/s12011-017-1142-8 28856594

[phy270118-bib-0075] Monzel, A. S. , Enríquez, J. A. , & Picard, M. (2023). Multifaceted mitochondria: Moving mitochondrial science beyond function and dysfunction. Nature Metabolism, 5, 546–562. 10.1038/s42255-023-00783-1 PMC10427836 37100996

[phy270118-bib-0076] Moreno‐Loshuertos, R. , Marco‐Brualla, J. , Meade, P. , Soler‐Agesta, R. , Enriquez, J. A. , & Fernández‐Silva, P. (2023). How hot can mitochondria be? Incubation at temperatures above 43°C induces the degradation of respiratory complexes and supercomplexes in intact cells and isolated mitochondria. Mitochondrion, 69, 83–94. 10.1016/j.mito.2023.02.002 36764502

[phy270118-bib-0077] Münzel, T. , Hahad, O. , Sørensen, M. , Lelieveld, J. , Duerr, G. D. , Nieuwenhuijsen, M. , & Daiber, A. (2021). Environmental risk factors and cardiovascular diseases: A comprehensive expert review. Cardiovascular Research, 118, 2880–2902. 10.1093/cvr/cvab316 PMC9648835 34609502

[phy270118-bib-0078] Naidoo, G. , & Naidoo, K. (2016). Uptake of polycyclic aromatic hydrocarbons and their cellular effects in the mangrove Bruguiera gymnorrhiza. Marine Pollution Bulletin, 113, 193–199. 10.1016/j.marpolbul.2016.09.012 27634737

[phy270118-bib-0080] Neikirk, K. , Kabugi, K. , Mungai, M. , Kula, B. , Smith, N. , & Hinton, A. O. (2024). Ethnicity‐related differences in mitochondrial regulation by insulin stimulation in diabetes. Journal of Cellular Physiology, 239, e31317. 10.1002/jcp.31317 38775168 PMC11324399

[phy270118-bib-0081] Neikirk, K. , Lopez, E.‐G. , Marshall, A. G. , Alghanem, A. , Krystofiak, E. , Kula, B. , Smith, N. , Shao, J. , Katti, P. , & Hinton, A. O. (2023). Call to action to properly utilize electron microscopy to measure organelles to monitor disease. European Journal of Cell Biology, 102(4), 151365. 37864884 10.1016/j.ejcb.2023.151365

[phy270118-bib-0082] Neikirk, K. , Stephens, D. C. , Beasley, H. K. , Marshall, A. G. , Gaddy, J. A. , Damo, S. M. , & Hinton, A. O. (2024). Considerations for developing mitochondrial transplantation techniques for individualized medicine. BioTechniques, 76, 125–134. 10.2144/btn-2023-0072 38420889

[phy270118-bib-0083] Neikirk, K. , Vue, Z. , Katti, P. , Rodriguez, B. I. , Omer, S. , Shao, J. , Christensen, T. , Garza Lopez, E. , Marshall, A. , Palavicino‐Maggio, C. B. , Ponce, J. , Alghanem, A. F. , Vang, L. , Barongan, T. , Beasley, H. K. , Rodman, T. , Stephens, D. , Mungai, M. , Correia, M. , … Hinton, A. O., Jr. (2023). Systematic transmission electron microscopy‐based identification and 3D reconstruction of cellular degradation machinery. Advanced Biology, 7, 2200221. 10.1002/adbi.202200221 PMC13150769 36869426

[phy270118-bib-0084] Nichtová, Z. , Fernandez‐Sanz, C. , De La Fuente, S. , Yuan, Y. , Hurst, S. , Lanvermann, S. , Tsai, H.‐Y. , Weaver, D. , Baggett, A. , Thompson, C. , Bouchet‐Marquis, C. , Várnai, P. , Seifert, E. L. , Dorn, G. W. , Sheu, S.‐S. , & Csordás, G. (2023). Enhanced mitochondria‐SR tethering triggers adaptive cardiac muscle remodeling. Circulation Research, 132, e171–e187. 10.1161/CIRCRESAHA.122.321833 37057625 PMC10213149

[phy270118-bib-0085] Parisi, F. , Fenizia, C. , Introini, A. , Zavatta, A. , Scaccabarozzi, C. , Biasin, M. , & Savasi, V. (2023). The pathophysiological role of estrogens in the initial stages of pregnancy: Molecular mechanisms and clinical implications for pregnancy outcome from the periconceptional period to end of the first trimester. Human Reproduction Update, 29, 699–720. 10.1093/humupd/dmad016 37353909 PMC10628507

[phy270118-bib-0086] Patterson Jr Donald, G. , Wong, L.‐Y. , Turner, W. E. , Caudill, S. P. , DiPietro, E. S. , McClure, P. C. , Cash, T. P. , Osterloh, J. D. , Pirkle, J. L. , Sampson, E. J. , & Needham, L. L. (2009). Levels in the U.S. population of those persistent organic pollutants (2003–2004) included in the Stockholm convention or in other long‐range transboundary air pollution agreements. Environmental Science & Technology, 43, 1211–1218. 10.1021/es801966w 19320182

[phy270118-bib-0087] Peng, C. , Sanchez‐Guerra, M. , Wilson, A. , Mehta, A. J. , Zhong, J. , Zanobetti, A. , Brennan, K. , Dereix, A. E. , Coull, B. A. , Vokonas, P. , Schwartz, J. , & Baccarelli, A. A. (2017). Short‐term effects of air temperature and mitochondrial DNA lesions within an older population. Environment International, 103, 23–29. 10.1016/j.envint.2017.03.017 28351767 PMC5849241

[phy270118-bib-0088] Polkowska, Ż. , Kot, A. , Wiergowski, M. , Wolska, L. , Wołowska, K. , & Namieśnik, J. (2000). Organic pollutants in precipitation: Determination of pesticides and polycyclic aromatic hydrocarbons in Gdańsk, Poland. Atmospheric Environment, 34, 1233–1245. 10.1016/S1352-2310(99)00180-6

[phy270118-bib-0089] Poznyak, A. V. , Ivanova, E. A. , Sobenin, I. A. , Yet, S.‐F. , & Orekhov, A. N. (2020). The role of mitochondria in cardiovascular diseases. Biology, 9, 137. 10.3390/biology9060137 32630516 PMC7344641

[phy270118-bib-0090] Pribylova, P. , Kares, R. , Boruvkova, J. , Cupr, P. , Prokes, R. , Kohoutek, J. , Holoubek, I. , & Klanova, J. (2012). Levels of persistent organic pollutants and polycyclic aromatic hydrocarbons in ambient air of central and Eastern Europe. Atmospheric Pollution Research, 3, 494–505. 10.5094/APR.2012.057

[phy270118-bib-0091] Pronsato, L. , Milanesi, L. , & Vasconsuelo, A. (2020). Testosterone induces up‐regulation of mitochondrial gene expression in murine C2C12 skeletal muscle cells accompanied by an increase of nuclear respiratory factor‐1 and its downstream effectors. Molecular and Cellular Endocrinology, 500, 110631. 10.1016/j.mce.2019.110631 31676390

[phy270118-bib-0092] Rainey, N. E. , Saric, A. , Leberre, A. , Dewailly, E. , Slomianny, C. , Vial, G. , Zeliger, H. I. , & Petit, P. X. (2017). Synergistic cellular effects including mitochondrial destabilization, autophagy and apoptosis following low‐level exposure to a mixture of lipophilic persistent organic pollutants. Scientific Reports, 7, 4728. 10.1038/s41598-017-04654-0 28680151 PMC5498599

[phy270118-bib-0093] Rajagopalan, S. , & Landrigan, P. J. (2021). Pollution and the heart. New England Journal of Medicine, 385, 1881–1892. 10.1056/NEJMra2030281 34758254

[phy270118-bib-0094] Ray, R. R. (2016). Adverse hematological effects of hexavalent chromium: An overview. Interdisciplinary Toxicology, 9, 55–65. 10.1515/intox-2016-0007 28652847 PMC5458105

[phy270118-bib-0095] Reddam, A. , McLarnan, S. , & Kupsco, A. (2022). Environmental chemical exposures and mitochondrial dysfunction: A review of recent literature. Current Environmental Health Reports, 9, 631–649. 10.1007/s40572-022-00371-7 35902457 PMC9729331

[phy270118-bib-0096] Resende, R. , Fernandes, T. , Pereira, A. C. , Marques, A. P. , & Pereira, C. F. (2022). Endoplasmic reticulum‐mitochondria contacts modulate reactive oxygen species‐mediated signaling and oxidative stress in brain disorders: The key role of sigma‐1 receptor. Antioxidants & Redox Signaling, 37, 758–780. 10.1089/ars.2020.8231 35369731

[phy270118-bib-0097] Sagheer, U. , Sadeer, A.‐K. , Abohashem, S. , Phillips, C. T. , Rana, J. S. , Bhatnagar, A. , Gulati, M. , Rajagopalan, S. , & Kalra, D. K. (2024). Environmental pollution and cardiovascular disease. JACC: Advances, 3, 100805. 10.1016/j.jacadv.2023.100805 38939394 PMC11198458

[phy270118-bib-0098] Sarma, S. N. , Blais, J. M. , & Chan, H. M. (2017). Neurotoxicity of alkylated polycyclic aromatic compounds in human neuroblastoma cells. Journal of Toxicology and Environmental Health, Part A, 80, 285–300. 10.1080/15287394.2017.1314840 28598261

[phy270118-bib-0099] Seiber, J. N. , & Cahill, T. M. (2022). Pesticides, organic contaminants, and pathogens in air: chemodynamics, health effects, sampling, and analysis. Taylor & Francis.

[phy270118-bib-0100] Shah, A. S. , Langrish, J. P. , Nair, H. , McAllister, D. A. , Hunter, A. L. , Donaldson, K. , Newby, D. E. , & Mills, N. L. (2013). Global association of air pollution and heart failure: A systematic review and meta‐analysis. The Lancet, 382, 1039–1048. 10.1016/S0140-6736(13)60898-3 PMC3809511 23849322

[phy270118-bib-0101] Soares, S. S. , Martins, H. , Gutiérrez‐Merino, C. , & Aureliano, M. (2008). Vanadium and cadmium in vivo effects in teleost cardiac muscle: Metal accumulation and oxidative stress markers. Comparative Biochemistry and Physiology Part C: Toxicology & Pharmacology, 147, 168–178. 10.1016/j.cbpc.2007.09.003 17920336

[phy270118-bib-0102] Sun, D. , & Yang, F. (2017). Metformin improves cardiac function in mice with heart failure after myocardial infarction by regulating mitochondrial energy metabolism. Biochemical and Biophysical Research Communications, 486, 329–335. 10.1016/j.bbrc.2017.03.036 28302481

[phy270118-bib-0103] Sun, M. , Ding, R. , Ma, Y. , Sun, Q. , Ren, X. , Sun, Z. , & Duan, J. (2021). Cardiovascular toxicity assessment of polyethylene nanoplastics on developing zebrafish embryos. Chemosphere, 282, 131124. 10.1016/j.chemosphere.2021.131124 34374342

[phy270118-bib-0104] Szabo, L. , Cummins, N. , Paganetti, P. , Odermatt, A. , Papassotiropoulos, A. , Karch, C. , Götz, J. , Eckert, A. , & Grimm, A. (2023). ER‐mitochondria contacts and cholesterol metabolism are disrupted by disease‐associated tau protein. EMBO Reports, 24, e57499. 10.15252/embr.202357499 37401859 PMC10398652

[phy270118-bib-0105] Tanner, C. M. , Kamel, F. , Ross, G. W. , Hoppin, J. A. , Goldman, S. M. , Korell, M. , Marras, C. , Bhudhikanok, G. S. , Kasten, M. , Chade, A. R. , Comyns, K. , Richards, M. B. , Meng, C. , Priestley, B. , Fernandez, H. H. , Cambi, F. , Umbach, D. M. , Blair, A. , Sandler, D. P. , & Langston, J. W. (2011). Rotenone, paraquat, and Parkinson's disease. Environmental Health Perspectives, 119, 866–872. 10.1289/ehp.1002839 21269927 PMC3114824

[phy270118-bib-0106] Terzioglu, M. , Veeroja, K. , Montonen, T. , Ihalainen, T. O. , Salminen, T. S. , Bénit, P. , Rustin, P. , Chang, Y.‐T. , Nagai, T. , & Jacobs, H. T. (2023). Mitochondrial temperature homeostasis resists external metabolic stresses. eLife, 12, RP89232. 10.7554/eLife.89232 38079477 PMC10712956

[phy270118-bib-0107] Toro‐Urrego, N. , Garcia‐Segura, L. M. , Echeverria, V. , & Barreto, G. E. (2016). Testosterone protects mitochondrial function and regulates neuroglobin expression in astrocytic cells exposed to glucose deprivation. Frontiers in Aging Neuroscience, 8, 152. 10.3389/fnagi.2016.00152 27445795 PMC4921852

[phy270118-bib-0108] Tostes, R. C. , Carneiro, F. S. , Carvalho, M. H. C. , & Reckelhoff, J. F. (2016). Reactive oxygen species: Players in the cardiovascular effects of testosterone. American Journal of Physiology. Regulatory, Integrative and Comparative Physiology, 310, R1–R14. 10.1152/ajpregu.00392.2014 26538238 PMC4796634

[phy270118-bib-0109] Touyz, R. M. , Rios, F. J. , Alves‐Lopes, R. , Neves, K. B. , Camargo, L. L. , & Montezano, A. C. (2020). Oxidative stress: A unifying paradigm in hypertension. Canadian Journal of Cardiology, 36, 659–670. 10.1016/j.cjca.2020.02.081 32389339 PMC7225748

[phy270118-bib-0110] Uusitalo, A. L. T. , Vanninen, E. , Levälahti, E. , Battié, M. C. , Videman, T. , & Kaprio, J. (2007). Role of genetic and environmental influences on heart rate variability in middle‐aged men. American Journal of Physiology. Heart and Circulatory Physiology, 293, H1013–H1022. 10.1152/ajpheart.00475.2006 17400723

[phy270118-bib-0111] van der Plaat, D. A. , de Jong, K. , de Vries, M. , van Diemen, C. C. , Nedeljković, I. , Amin, N. , Kromhout, H. , Consortium BIOS , Vermeulen, R. , Postma, D. S. , van Duijn, C. M. , Boezen, H. M. , & Vonk, J. M. (2018). Occupational exposure to pesticides is associated with differential DNA methylation. Occupational and Environmental Medicine, 75, 427–435. 10.1136/oemed-2017-104787 29459480 PMC5969365

[phy270118-bib-0112] Vasiljevic, T. , & Harner, T. (2021). Bisphenol A and its analogues in outdoor and indoor air: Properties, sources and global levels. Science of the Total Environment, 789, 148013. 10.1016/j.scitotenv.2021.148013 34323825

[phy270118-bib-0114] Vincent, A. E. , Turnbull, D. M. , Eisner, V. , Hajnóczky, G. , & Picard, M. (2017). Mitochondrial Nanotunnels. Trends in Cell Biology, 27, 787–799. 10.1016/j.tcb.2017.08.009 28935166 PMC5749270

[phy270118-bib-0115] Vincent, A. E. , White, K. , Davey, T. , Philips, J. , Ogden, R. T. , Lawless, C. , Warren, C. , Hall, M. G. , Ng, Y. S. , Falkous, G. , Holden, T. , Deehan, D. , Taylor, R. W. , Turnbull, D. M. , & Picard, M. (2019). Quantitative 3D mapping of the human skeletal muscle mitochondrial network. Cell Reports, 26, 996–1009.e4. 10.1016/j.celrep.2019.01.010 30655224 PMC6513570

[phy270118-bib-0117] Vue, Z. , Garza‐Lopez, E. , Neikirk, K. , Vang, L. , Beasley, H. , Marshall, A. G. , Murray, S. A. , & Hinton, A., Jr. (2022). Mouse skeletal muscle decrease in the MICOS complex and altered mitochondrial networks with age. *BioRxiv* .

[phy270118-bib-0118] Vue, Z. , Neikirk, K. , Vang, L. , Garza‐Lopez, E. , Christensen, T. A. , Shao, J. , Lam, J. , Beasley, H. K. , Marshall, A. G. , Crabtree, A. , Anudokem, J. , Rodriguez, B. , Kirk, B. , Bacevac, S. , Barongan, T. , Shao, B. , Stephens, D. C. , Kabugi, K. , Koh, H.‐J. , … Hinton, A. (2023). Three‐dimensional mitochondria reconstructions of murine cardiac muscle changes in size across aging. American Journal of Physiology. Heart and Circulatory Physiology, 325(5), H965–H982. 37624101 10.1152/ajpheart.00202.2023 PMC10977873

[phy270118-bib-0119] Wang, M. , Zhao, J. , Wang, Y. , Mao, Y. , Zhao, X. , Huang, P. , Liu, Q. , Ma, Y. , Yao, Y. , Yang, Z. , Yuan, W. , Cui, W. , Payne, T. J. , & Li, M. D. (2020). Genome‐wide DNA methylation analysis reveals significant impact of long‐term ambient air pollution exposure on biological functions related to mitochondria and immune response. Environmental Pollution, 264, 114707. 10.1016/j.envpol.2020.114707 32388307

[phy270118-bib-0120] Wang, Y. , Li, S. , Yang, S. , Li, X. , Liu, L. , Ma, X. , Niu, D. , & Duan, X. (2022). Exposure to phenanthrene affects oocyte meiosis by inducing mitochondrial dysfunction and endoplasmic reticulum stress. Cell Proliferation, 56, e13335. 10.1111/cpr.13335 36125441 PMC9816937

[phy270118-bib-0121] Weinhouse, C. (2017). Mitochondrial‐epigenetic crosstalk in environmental toxicology. Toxicology, 391, 5–17. 10.1016/j.tox.2017.08.008 28855114 PMC5681427

[phy270118-bib-0122] Wen, Z.‐J. , Wei, Y.‐J. , Zhang, Y.‐F. , & Zhang, Y.‐F. (2023). A review of cardiovascular effects and underlying mechanisms of legacy and emerging per‐ and polyfluoroalkyl substances (PFAS). Archives of Toxicology, 97, 1195–1245. 10.1007/s00204-023-03477-5 36947184

[phy270118-bib-0123] Witkowska, D. , Słowik, J. , & Chilicka, K. (2021). Heavy metals and human health: Possible exposure pathways and the competition for protein binding sites. Molecules, 26, 6060. 10.3390/molecules26196060 34641604 PMC8511997

[phy270118-bib-0125] Wu, Q. , Cao, J. , Liu, X. , Zhu, X. , Huang, C. , Wang, X. , & Song, Y. (2024). Micro(nano)‐plastics exposure induced programmed cell death and corresponding influence factors. Science of the Total Environment, 921, 171230. 10.1016/j.scitotenv.2024.171230 38402958

[phy270118-bib-0126] Wu, S. , Lu, Q. , Wang, Q. , Ding, Y. , Ma, Z. , Mao, X. , Huang, K. , Xie, Z. , & Zou, M.‐H. (2017). Binding of FUN14 domain containing 1 with inositol 1,4,5‐trisphosphate receptor in mitochondria‐associated endoplasmic reticulum membranes maintains mitochondrial dynamics and function in hearts in vivo. Circulation, 136, 2248–2266. 10.1161/CIRCULATIONAHA.117.030235 28942427 PMC5716911

[phy270118-bib-0127] Xiang, D. , Liu, Y. , Zhou, S. , Zhou, E. , & Wang, Y. (2021). Protective effects of estrogen on cardiovascular disease mediated by oxidative stress. Oxidative Medicine and Cellular Longevity, 2021, 5523516. 10.1155/2021/5523516 34257804 PMC8260319

[phy270118-bib-0128] Yang, D. , Yang, Q. , Fu, N. , Li, S. , Han, B. , Liu, Y. , Tang, Y. , Guo, X. , Lv, Z. , & Zhang, Z. (2021). Hexavalent chromium induced heart dysfunction via Sesn2‐mediated impairment of mitochondrial function and energy supply. Chemosphere, 264, 128547. 10.1016/j.chemosphere.2020.128547 33049514

[phy270118-bib-0129] Yang, Q. , Han, B. , Xue, J. , Lv, Y. , Li, S. , Liu, Y. , Wu, P. , Wang, X. , & Zhang, Z. (2020). Hexavalent chromium induces mitochondrial dynamics disorder in rat liver by inhibiting AMPK/PGC‐1α signaling pathway. Environmental Pollution, 265, 114855. 10.1016/j.envpol.2020.114855 32474337

[phy270118-bib-0130] Zhang, L. , Yang, F. , Li, Y. , Cao, H. , Huang, A. , Zhuang, Y. , Zhang, C. , Hu, G. , Mao, Y. , Luo, J. , & Xing, C. (2021). The protection of selenium against cadmium‐induced mitophagy via modulating nuclear xenobiotic receptors response and oxidative stress in the liver of rabbits. Environmental Pollution, 285, 117301. 10.1016/j.envpol.2021.117301 34049137

[phy270118-bib-0131] Zhao, M. , Xie, J. , Zhang, J. , Zhao, B. , Zhang, Y. , Xue, J. , Zhang, R. , Zhang, R. , Wang, H. , Li, Y. , Ge, W. , & Zhou, X. (2024). Disturbance of mitochondrial dynamics led to spermatogenesis disorder in mice exposed to polystyrene micro‐ and nanoplastics. Environmental Pollution, 362, 124935. 10.1016/j.envpol.2024.124935 39260550

[phy270118-bib-0132] Zhao, X. , Li, X. , Wang, S. , Yang, Z. , Liu, H. , & Xu, S. (2021). Cadmium exposure induces mitochondrial pathway apoptosis in swine myocardium through xenobiotic receptors‐mediated CYP450s activation. Journal of Inorganic Biochemistry, 217, 111361. 10.1016/j.jinorgbio.2021.111361 33581611

[phy270118-bib-0133] Zhao, Y. , Chang, Y.‐H. , Ren, H.‐R. , Lou, M. , Jiang, F.‐W. , Wang, J.‐X. , Chen, M.‐S. , Liu, S. , Shi, Y.‐S. , Zhu, H.‐M. , & Li, J.‐L. (2024). Phthalates induce neurotoxicity by disrupting the Mfn2‐PERK axis‐mediated endoplasmic reticulum–mitochondria interaction. Journal of Agricultural and Food Chemistry, 72, 7411–7422. 10.1021/acs.jafc.3c07752 38390847

[phy270118-bib-0134] Zheng, F. , Aschner, M. , & Li, H. (2021). Evaluations of environmental pollutant‐induced mitochondrial toxicity using Caenorhabditis elegans as a model system. In X. Pan & B. Zhang (Eds.), Environmental toxicology and toxicogenomics: Principles, methods, and applications (pp. 33–46). Springer. 10.1007/978-1-0716-1514-0_3 34097259

[phy270118-bib-0135] Zhou, B. , & Tian, R. (2018). Mitochondrial dysfunction in pathophysiology of heart failure. The Journal of Clinical Investigation, 128, 3716–3726. 10.1172/JCI120849 30124471 PMC6118589

[phy270118-bib-0136] Ziegler, D. V. , Martin, N. , & Bernard, D. (2021). Cellular senescence links mitochondria‐ER contacts and aging. Communications Biology, 4, 1–14. 10.1038/s42003-021-02840-5 34819602 PMC8613202

[phy270118-bib-0137] Zolkipli‐Cunningham, Z. , & Falk, M. J. (2017). Clinical effects of chemical exposures on mitochondrial function. Toxicology, 391, 90–99. 10.1016/j.tox.2017.07.009 28757096 PMC6078194

